# Molecular identity of the lateral lemniscus nuclei in the adult mouse brain

**DOI:** 10.3389/fnana.2023.1098352

**Published:** 2023-03-09

**Authors:** Isabel M. García-Guillén, Pilar Aroca, Faustino Marín

**Affiliations:** Department of Human Anatomy and Psychobiology, Faculty of Medicine, Regional Campus of International Excellence “Campus Mare Nostrum”, Biomedical Research Institute of Murcia (IMIB-Pascual Parrilla), University of Murcia, Murcia, Spain

**Keywords:** auditory pathway, hearing pathway, cochlear nuclei, superior olive, inferior colliculus, gene expression

## Abstract

The dorsal (DLL), intermediate (ILL), and ventral (VLL) lateral lemniscus nuclei are relay centers in the central auditory pathway of the brainstem, commonly referred to as the lateral lemniscus nuclei (LLN). The LLN are situated in the prepontine and pontine hindbrain, from rhombomeres 1 to 4, extending from the more rostral DLL to the caudal VLL, with the ILL lying in between. These nuclei can be distinguished morphologically and by topological and connectivity criteria, and here, we set out to further characterize the molecular nature of each LLN. We searched *in situ* hybridization studies in the Allen Mouse Brain Atlas for genes differentially expressed along the rostrocaudal axis of the brainstem, identifying 36 genes from diverse functional families expressed in the LLN. Available information in the databases indicated that 7 of these 36 genes are either associated with or potentially related to hearing disorders. In conclusion, the LLN are characterized by specific molecular profiles that reflect their rostrocaudal organization into the three constituent nuclei. This molecular regionalization may be involved in the etiology of some hearing disorders, in accordance with previous functional studies of these genes.

## 1. Introduction

The lateral lemniscus nuclei (LLN) constitute a complex of three independent nuclei in the main auditory pathway of sound perception and spatial sound localization. The LLN transmits signals from lower auditory brainstem nuclei like the cochlear nuclear complex (CN) and the superior olivary complex (SOC), to higher auditory structures like the inferior colliculus (IC) in the midbrain or the medial geniculate nucleus in the thalamus (Frisina and Walton, 2001; Grothe et al., 2010).

The LLN is comprised of the dorsal (DLL), intermediate (ILL) and ventral (VLL) nuclei, each of which is embedded within the fibers of the lateral lemniscus tract. These subnuclei are located in the prepontine and pontine hindbrain, with both the DLL and ILL lying within rhombomere (r) 1, while the VLL extends from r2 to r4 (Di Bonito et al., 2013; Altieri et al., 2016; Di Bonito and Studer, 2017).

Some studies consider that the LLN in rodents is composed of two nuclei, the DLL and the VLL (Campos et al., 2001; Riquelme et al., 2001; Koch et al., 2004), which participate in the processing of binaural and monoaural afferences, respectively (Merchán and Berbel, 1996; Malmierca and Ryugo, 2012; Malmierca, 2015). Nevertheless, the VLL described by these authors can be further subdivided into ILL and VLL according to their patterns of connectivity to other central auditory nuclei and other distinguishable features (Glendenning et al., 1981; Nayagam et al., 2006; Kelly et al., 2009; Ito et al., 2011; Felmy, 2019).

The three LLN also display distinct neurotransmitter phenotypes in rodents, whereby the DLL and the VLL are mainly inhibitory, with strong expression of the Vesicular Inhibitory Amino Acid Transporter (VIAAT/VGAT/*Slc32a1*), a marker of both GABAergic and glycinergic neurons, and very weak expression of the Vesicular Glutamate Transporters (VGluT1/*Slc17a7*, VGluT2/*Slc17a6*) that are markers of glutamatergic neurons (Ito et al., 2011; Di Bonito and Studer, 2017). In addition, the DLL and VLL both express the Gad67/Gad1 (Di Bonito et al., 2013, 2017; Di Bonito and Studer, 2017) and Gad65/*Gad2* GABAergic markers (Riquelme et al., 2001), and the GABA_*A*_ receptor subunits *Gabra1, Gabrb3*, and *Gabrg2*, while *Gabrd* is also expressed in the VLL (Campos et al., 2001). By contrast, the ILL is mainly excitatory with the majority of neurons expressing VGluT1 and/or VGluT2, and only a few of their neurons expressing VIAAT (Ito et al., 2011; Di Bonito and Studer, 2017; Di Bonito et al., 2017). In terms of other genes related to neurotransmission, *Pnoc* (Prepronociceptin) expression has been detected in the DLL (Boom et al., 1999).

Other neural-related molecules are also expressed regionally within the LLN. Calbindin (*Calb*) immunoreactivity (Calbindin-D28K) has been detected in the DLL and VLL of adult rats (Friauf, 1994), although the expression of this molecule shows significant developmental and interspecies variation (see Discussion). On the other hand, the *Hcn1* and *Kcnq4* potassium channels are expressed in VLL, and in the VLL and ILL, respectively (Kharkovets et al., 2000; Koch et al., 2004). There is also graded expression of EphA-ephrinB interacting proteins in the lemniscal complex, which may be related to tonotopic maps (Wallace et al., 2016). In addition, the synaptic protein *Nrxn3* is expressed significantly in the DLL (Uchigashima et al., 2019).

The regional expression of several transcription factors has been described within the adult LLN and while *Gata3* is only expressed in the VLL (Di Bonito et al., 2013, 2017), *Pax7* and *Foxp2* are markers of the DLL (Stoykova and Gruss, 1994; Campbell et al., 2009; Di Bonito and Studer, 2017).

Therefore, the LLN nuclear complex is a structure in which distinct molecular markers are specifically expressed, such as transcription factors, neurotransmitters and other signaling molecules. To further characterize the molecular regional identities within this complex, aiming as well to shed light on the aforementioned issue about its subdivision into either two or three nuclei, we set out to analyzed the expression of genes from different families. As such, we initially searched for genes that are differentially expressed in the adult LLN using the Allen Mouse Brain Atlas (AMBA) database (Lein et al., 2007). Additionally, we looked for the possible clinical relevance for the auditory system of the identified genes, searching in mouse and human databases. Through this analysis we identified 36 genes expressed within the adult LLN, characterizing their expression relative to the specific nuclei that make up this structure: the DLL, ILL and VLL. The genes we found belong to different functional families, including genes known to cause hearing disorders.

## 2. Materials and methods

### 2.1. Delimitation of LLN

Prior to performing the gene expression analysis, we identified the limits of the LLN in sagittal and coronal sections through the specific labeling of the lateral lemniscus fibers, correlated with Nissl-stained preparations in equivalent planes and following the description of these structures in the literature (Glendenning et al., 1981; Franklin and Paxinos, 2007; Paxinos and Franklin, 2019).

The lateral lemniscus fibers were labeled in experiments using a transgenic reporter expressed by ascending cochlear nuclei axons (from the Gene Expression Nervous System Atlas-GENSAT- database)^1^ (Gong et al., 2003) or through dye injection (from the Allen Mouse Brain Connectivity Atlas -AMBCA- database)^2^ (Oh et al., 2014).

The GENSAT database provides images from transgenic mouse lines carrying EGFP as a reporter for the expression of different genes, presenting brain sections processed for immunohistochemistry against EGFP that normally labels the soma and fibers of positive neurons. We searched for genes expressed in the cochlear nuclei whose axons contribute to the lateral lemniscus tract, reaching the IC. One specimen from the *Doc2g* reporter line was selected, so that we downloaded and cropped the images to show our region of interest.

In addition, we utilized the Allen Mouse Brain Connectivity Atlas database, a high-resolution map of neural connections in the mouse brain. This atlas includes data from viral tracer injections (recombinant adeno-associated viruses -rAAV) into specific brain regions, such that the axonal projections from the infected neurons can be visualized through the expression of EGFP. We studied tracer injection into the ventral cochlear nucleus, allowing visualization of a large part of the lateral lemniscus fibers that reach the IC.

### 2.2. Mining the Allen brain database

For the core of this work, we searched a list of 4,063 genes in the Allen Mouse Brain Atlas database (AMBA) for which *in situ* hybridization (ISH) data were available from series of sagittal and coronal sections at P56.^3^ These data consisted of brightfield microphotographs and their counterparts with color-coding of their relative strength of expression. In the brightfield images, the positive cells accumulated a blue precipitate according to standard ISH protocols, while in the color-coded images the intensity of expression ranged from blue (weak expression), through green (medium intensity) to red (strong expression). We analyzed these image series to select genes with regionalized expression within the LLN coherently reproduced in both sagittal and coronal planes. As a result of this screening, we identified 36 genes (Table 1).

**TABLE 1:** Summary of the expression of each gene in the lateral lemniscus nuclei.

		DLL	ILL	VLL	References
Figure 2	**Hpse**	+ +			
Figure 2	**Pax7**	+ +			Stoykova and Gruss, 1994; Di Bonito and Studer, 2017
Figure 2	**Pnoc**	+ +			Boom et al., 1999
	**Cgnl1**	+ +			
	**Fam163b**	+ +			
	**Rapgef5**	+ +			
Figure 3	**Fstl1**	+		+	
Figure 3	**Gabra5**	+ +		+	
Figure 3	**Gad1**	+ +		+ +	Di Bonito and Studer, 2017; Ito et al., 2018; and references therein
Figure 4	**Inhbb**	+		+ +	
Figure 4	**Rreb1**	+ +		+	
	**Gad2**	+ +		+ +	Riquelme et al., 2001
	**Slc32a1**	+ +		+ +	Ito et al., 2011; Di Bonito and Studer, 2017; and references therein
Figure 5	**Foxp2**		+ +		Campbell et al., 2009 *
Figure 5	**Htr1a**		+		Daval et al., 1987 ^†^
Figure 5	**Nrn1**		+ +		
Figure 5	**Slc17a6**		+ +		Di Bonito and Studer, 2017; Ito et al., 2018; and references therein
Figure 6	**Hcn1**		+ +	+ +	Koch et al., 2004 *
Figure 6	**Igfbp5**		+ +	+ +	Bondy and Lee, 1993 ^†^
Figure 6	**Tnnt1**		+	++	
	**Cacng5**		+ +	+ +	
	**Cd24a**		+	++	Stott et al., 2017 ^†^
	**Crtac1**		+	+ +	
	**Kcnh7**		+ +	+ +	
	**Kcnq4**		+	+ +	Kharkovets et al., 2000
	**Meis2**		+ +	++	
	**Mgat5b**		+	+	
	**Prkcd**		+	+	
	**Rims3**		+ +	+ +	
Figure 7	**Calb1**			+ +	Friauf, 1994 *
Figure 7	**Doc2g**			+ +	
Figure 7	**Tmem215**			+ +	
	**Gata3**			+	Di Bonito et al., 2013; Di Bonito and Studer, 2017; and references therein
	**Kit**			+	Hirota et al., 1992 ^†^
	**Mgll**			+	
	**Sfrp1**			+	

+, ++, indicate two semiquantitative relative levels of expression from low to high.

*The previously published pattern does not fully coincide with the present data.

^†^These articles mention gene or protein expression in LLN, not specifying in which nuclei.

The images that included the whole LLN were downloaded and cropped to show the region of interest. In the figures, the images from parasagittal sections are orientated with the rostral end to the left, while those from coronal sections show the left side of the original image, with the midline on the right.

Images of some selected genes from embryonic (E18.5) and postnatal stages (P4, P28) were also downloaded from the Allen Developing Mouse Brain Atlas (ADMBA)^4^ (Thompson et al., 2014) (see section “3. Results”), following the same procedure.

The list of the selected genes, together with the references of their respective experiments and downloaded images from each database, are indicated in Supplementary Table 1.

### 2.3. Online Mendelian Inheritance in Man (OMIM) and Mouse Genome Informatics (MGI) search

We assessed the OMIM^5^ database and the literature to see if any of the 36 selected genes have been associated with human deafness, hearing disorders or auditory specific phenotypes.

In addition, we searched OMIM for the critical human chromosomic regions or loci that are associated with such dysfunctions. Subsequently, we retrieved the gene list of these loci from Ensemble/BioMart^6^ to check if it included any of the 36 genes selected.

We also compared our selected genes with the list of genes with mutations that cause deafness or hearing dysfunction in mice retrieved from MGI.^7^

## 3. Results

### 3.1. Regional identification of the lateral lemniscus nuclei

**FIGURE 1:**
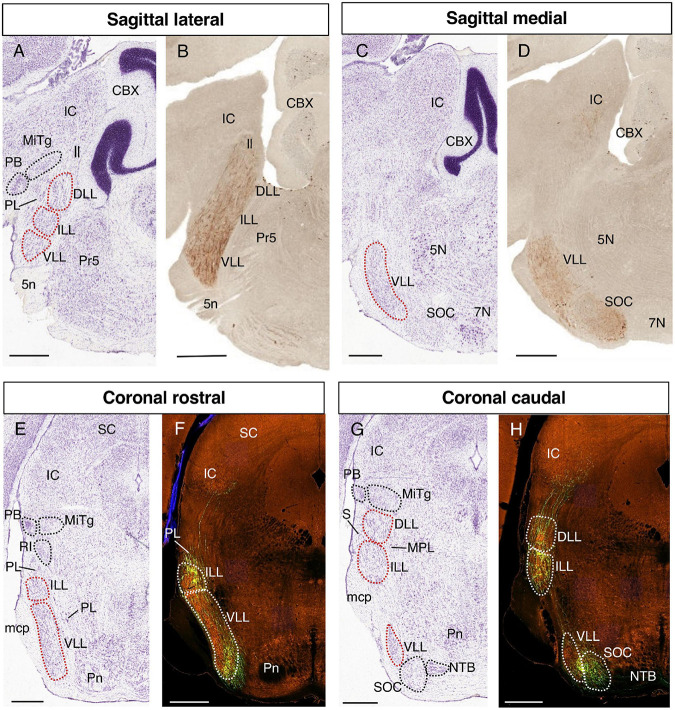
Identification of the LLN. Representative parasagittal **(A–D)** and coronal **(E–H)** sections centered on the LLN in the rostral hindbrain, corresponding to Nissl-stained sections from the Allen Reference Atlases **(A,C,E,G)** a *Doc2g*-EGFP reporter line from GENSAT **(B,D)** and EGFP viral transfection of cochlear fibers from the Allen Mouse Brain Connectivity Atlas **(F,H)**. In this and following figures, the images from parasagittal sections are oriented with the rostral end to the left, while those from coronal sections are details of the left side of the original image, with the midline to the right. All the images are centered on the LLN in the rostral hindbrain and the orientation of each image (sagittal/coronal, lateral/medial, rostral/caudal) is indicated above. The abbreviations used are as specified in the main text. Scale bars = 600 μm.

The LLN (formed by the DLL, ILL, and VLL) can be observed in lateral sagittal sections (Figures 1A, B) as an uninterrupted cellular column rostral to the principal nucleus of the trigeminal column (Pr5), and caudal to isthmic nuclei such as the parabigeminal nucleus (PB) and the microcellular tegmental nucleus (MiTg). The LLN is surrounded by the paralemniscal nucleus (PL), and it is separated from the IC dorsally by the MiTg and the lemniscus fibers (ll), while ventrally the fibers correspond to the trigeminal nerve root (5n) or to the middle cerebellar peduncle (mcp). In medial sagittal sections (Figures 1C, D), the VLL can be seen rostral to the SOC and the facial nucleus (7N). When considering the rostrocaudal order of the LLN, the DLL is evident at the rostral end of this complex in lateral sagittal sections, while the VLL is found caudally in the more medial sections, abutting the periolivary and olivary complex.

In representative coronal sections, the VLL and ILL can be observed at rostral levels (Figures 1E, F), and the DLL and ILL at caudal levels, together with a small portion of VLL (Figures 1G, H). In the aforementioned rostral sections (Figures 1E, F), the ILL is surrounded by the paralemniscal nucleus (PL), while rostral structures appear more dorsally, such as the retroisthmic nucleus (RI), the MiTg and the PB. Fibers from the middle cerebellar peduncle (mcp) can be found lateral to the ILL and VLL.

In a coronal caudal section containing the three nuclei (Figures 1G, H), the DLL is separated from the IC by the MiTg and the PB. The DLL and ILL are superficial, only separated from the pial surface by the sagulum (S). The medial paralemniscal nucleus (MPL) lies medial to the DLL and ILL, while part of the VLL is found ventrally, followed ventromedially by the SOC and the nucleus of the trapezoid body (NTB).

It is noteworthy that while the VLL and DLL are the most caudal and rostral nuclei of the LLN, they nevertheless appear in rostral and caudal coronal sections, respectively (Franklin and Paxinos, 2007; Paxinos and Franklin, 2019; present data). The relative positioning of these structures is due to the curvature of the rostrocaudal axis at the pontine flexure. This developmental deformation implies that in coronal sections of the prepontine/pontine regions, the rostral to caudal axis appears from the top to bottom of the image (see for example Puelles, 2001; Puelles et al., 2013; respectively their Figures 6, 1E).

### 3.2. Genes with regional expression in the lateral lemniscus nuclei

**FIGURE 2:**
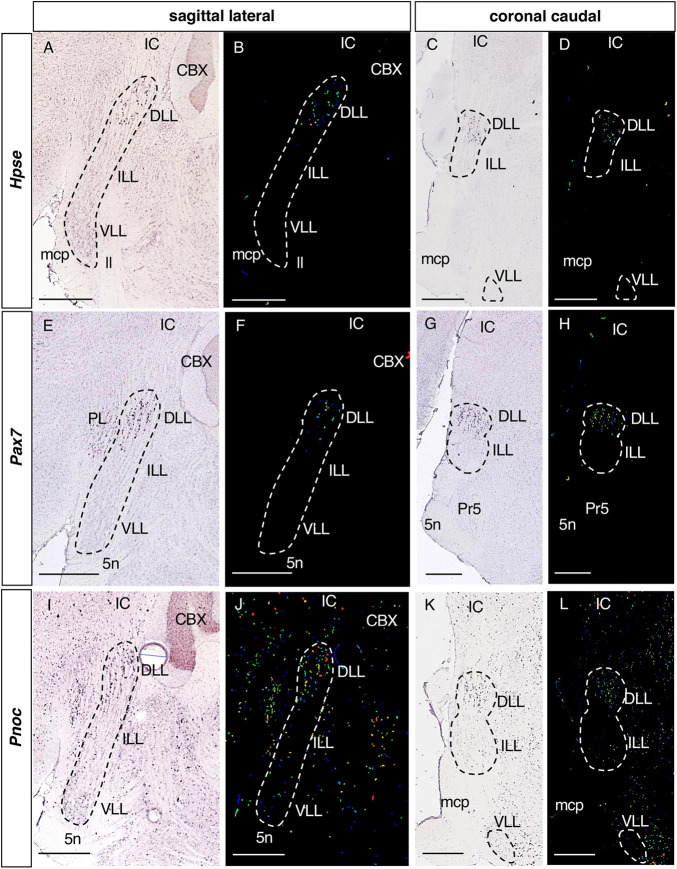
Expression of regionalized genes in the DLL. Brightfield **(A,C,E,G,I,K)** and color-coded **(B,D,F,H,J,L)** images of sections from P56 brains processed to detect *Hpse* **(A–D)**, *Pax7* **(E–H)** and *Pnoc* **(I–L)** expression. These three genes are expressed in the DLL, while they are absent from the ILL and VLL, except for a few *Pnoc* positive cells in the latter two nuclei **(I–L)**. In this and following figures, the gene name is given on the left. Scale bars = 600 μm.

**FIGURE 3:**
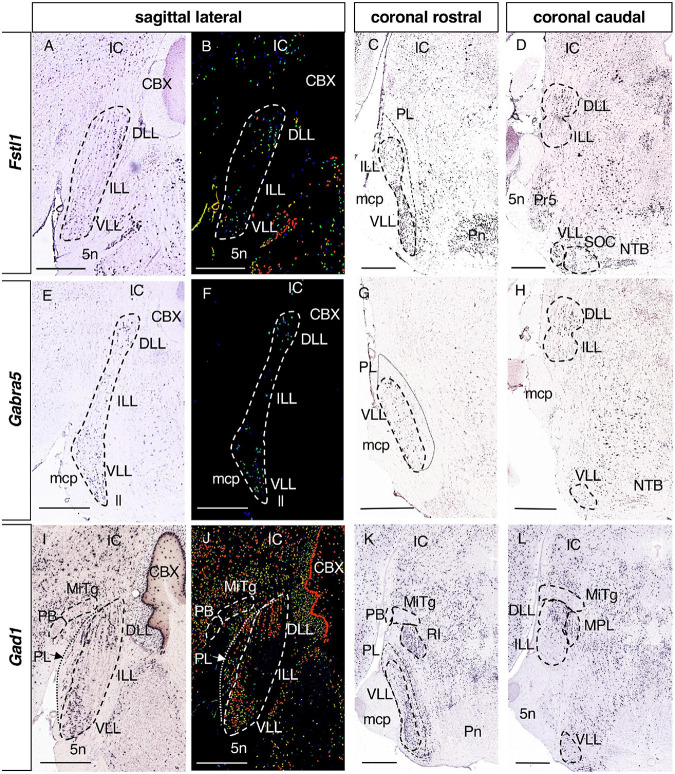
Expression of regionalized genes in the DLL and VLL (I). Brightfield **(A,C,D,E,G,H,I,K,L)** and color-coded **(B,F,J)** images of sections from P56 brains processed to the detect *Fstl1* **(A–D)**, *Gabra5* **(E–H)** and *Gad1* **(I–L)** expression. These three genes are expressed in the DLL and the VLL, while they are absent from the ILL. Scale bars = 600 μm.

**FIGURE 4:**
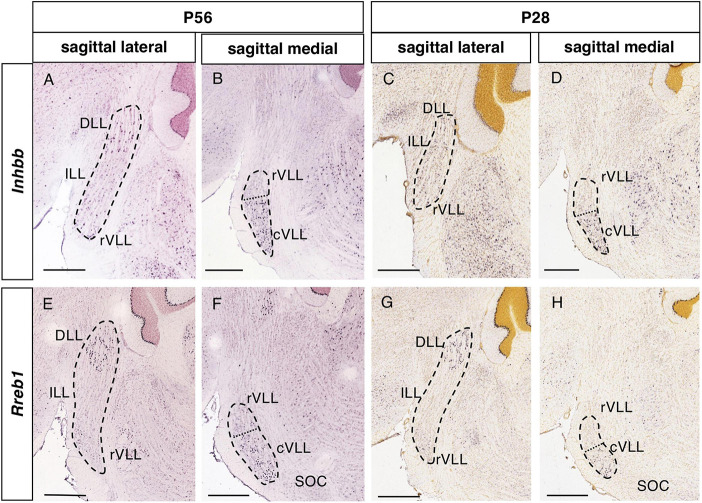
Expression of regionalized genes in the DLL and VLL (II). Brightfield images of sections from P56 **(A,B,E,F)** and P28 **(C,D,G,H)** brains processed to detect *Inhbb* **(A–D)** and *Rreb1* **(E–H)** expression. These genes are expressed in the DLL and the VLL, with no expression in the ILL. The expression in the VLL is regionalized, since there are few or no labeled cells in the lateral planes **(A,C,E,G)**, while expression is notable in more medial planes **(B,D,F,H)**, especially in the most caudal aspect of the VLL. The dotted lines in **(B,D,F,H)** indicate the limit between the apparent rostral (rVLL) and caudal (cVLL) subdivision of this nucleus based on these expression patterns. Scale bars = 600 μm.

**FIGURE 5:**
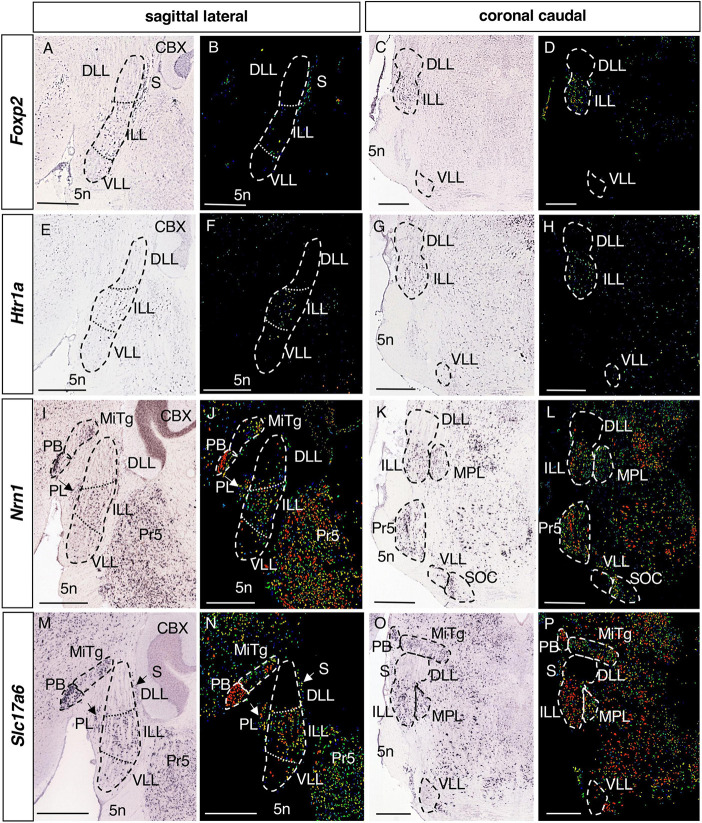
Expression of regionalized genes in the ILL. Brightfield **(A,C,E,G,I,K,M,O)** and color-coded **(B,D,F,H,J,L,N,P)** images of sections from P56 brains processed to detect *Foxp2* **(A–D)**, *Htr1a* **(E–H)**, *Nrn1* **(I–L)** and *Slc17a6* **(M–P)** expression. The expression of these genes in the LLN is restricted to the ILL, except for a few scattered positive cells in the DLL and/or VLL. Scale bars = 600 μm.

**FIGURE 6:**
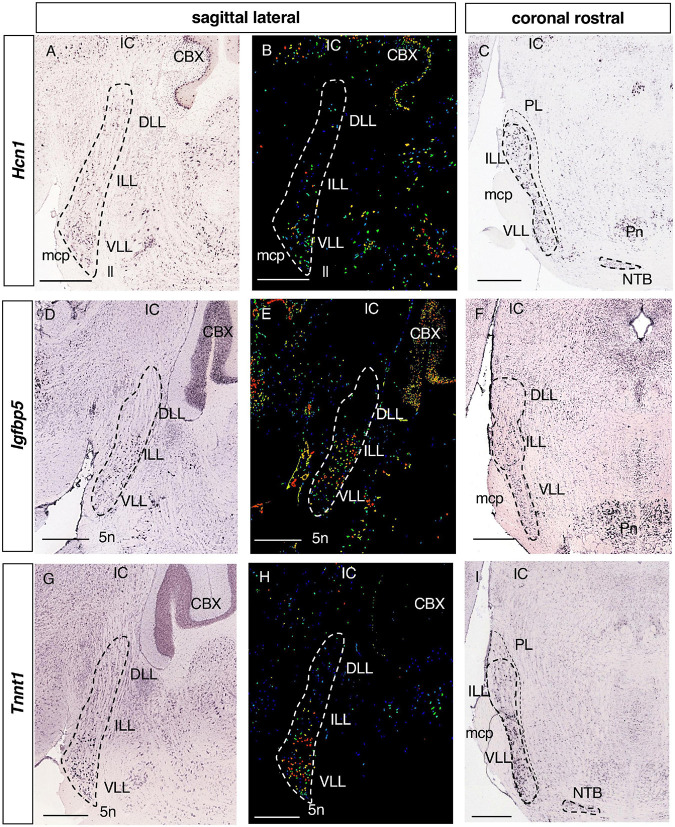
Expression of regionalized genes in the ILL and VLL. Brightfield **(A,C,D,F,G,I)** and color-coded **(B,E,H)** images of sections from P56 brains processed to detect *Hcn1* **(A–C)**, *Igfbp5* **(D–F)** and *Tnnt1* **(G–I)** expression. These genes are principally expressed in the ILL and VLL of the LLN. *Hcn1* and *Tnnt1* labeling is seen in some DLL cells **(A,B,G,H)**. Scale bars = 600 μm.

**FIGURE 7:**
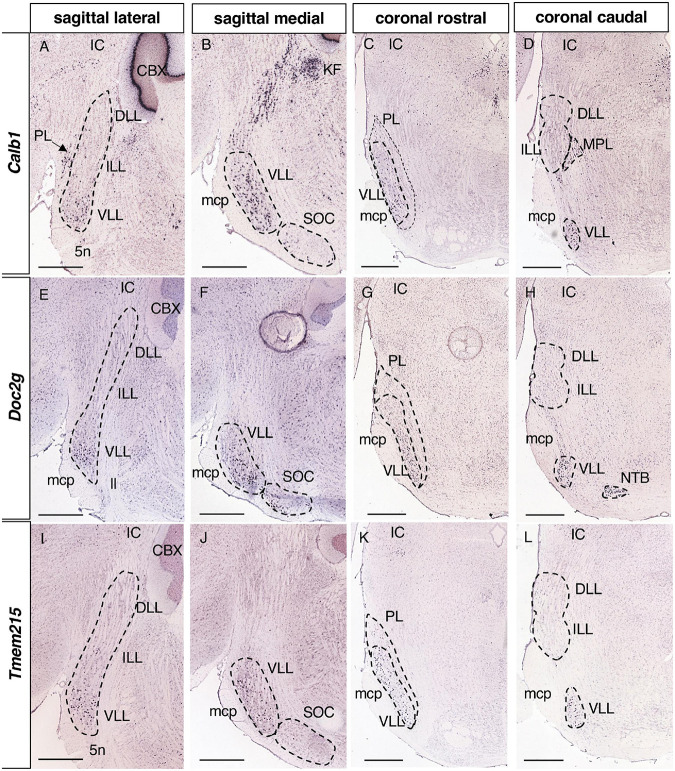
Expression of regionalized genes in the VLL. Brightfield images of sections from P56 brains processed to detect *Calb1* **(A–D)**, *Doc2g* **(E–H)** and *Tmem215* **(I–L)** expression. These three genes are expressed in the VLL, both in the lateral **(A,E,I)** and medial **(B,F,J)** planes, but not in the ILL or DLL **(A,D,E,H,I,L)**. Scale bars = 600 μm.

We searched for genes differentially expressed across LLN and identified 36 genes expressed in the different rostrocaudal subdivisions of the LLN, the DLL, ILL, and VLL. We grouped the genes with similar expression patterns based on their expression in a single LLN nuclei or in combinations of these (Table 1). As such, 6 genes were expressed the DLL alone (Figure 2), 7 genes in both the DLL and VLL (Figures 3, 4), 4 genes exclusively in the ILL (Figure 5), 12 genes in both the ILL and VLL (Figure 6), and 7 genes only in the VLL (Figure 7).

#### 3.2.1. Genes expressed in the DLL

Of the 36 genes identified in the screening, 6 are expressed exclusively in the DLL (Table 1) and not in the ILL or VLL: *Hpse*, Heparanase (Figures 2A–D); *Pax7*, Paired box 7 (Figures 2E–H); *Pnoc*, Prepronociceptin (Figures 2I–L); *Cgnl1*, Cingulin-like 1; *Fam163b*, Family with sequence similarity 163 member B; and *Rapgef5*, Rap guanine nucleotide exchange factor 5 (data not shown).

The expression of *Hpse* and *Pax7* (Figures 2A–H) in the DLL is very specific, with little or no expression in the surrounding structures. Conversely, *Pnoc* (Figures 2I–L) is also expressed in neighboring brainstem nuclei, like *Cgnl1, Fam163b*, and *Rapgef5* (data not shown).

#### 3.2.2. Genes expressed in the DLL and VLL

There were 8 genes expressed in both the DLL and VLL but not in the ILL (Table 1): *Fstl1*, Follistatin-like 1 (Figures 3A–D); *Gabra5*, Gamma-aminobutyric acid (GABA) A receptor subunit alpha 5 (Figures 3E–H); *Gad1*, Glutamate decarboxylase 1 (Figures 3I–L); *Inhbb*, Inhibin beta-B (Figures 4A–D); *Rreb1*, Ras responsive element binding protein 1 (Figures 4E–H); *Gad2*, Glutamic acid decarboxylase 2; and *Slc32a1*, Solute carrier family 32 (GABA vesicular transporter) member 1 (data not shown).

The expression of *Inhbb* and *Rreb1* in the VLL is restricted to a caudal portion, that is visible in medial sagittal sections abutting the SOC (Figures 4B, F, respectively). In light of this pattern, we tentatively subdivided the VLL into rostral and caudal subdivisions (rVLL and cVLL), in which the expression of these two genes was (cVLL) or was not (rVLL) detected. Given this particular distribution of these genes within the VLL, we assessed that it was also evident at other stages, i.e., P28 (Figures 4D, H).

#### 3.2.3. Genes expressed in the ILL

The expression of 4 genes is restricted to the ILL alone (Table 1): *Foxp2*, Forkhead box P2 (Figures 5A–D); *Htr1a*, 5-Hydroxytryptamine (serotonin) receptor 1A (Figures 5E–H); *Nrn1*, Neuritin 1 (Figures 5I–L) and *Slc17a6*, Solute carrier family 17 member 6 (Figures 5M–P). On the other hand, *Nrn1* and *Slc17a6* are also expressed in other neighboring structures, such as the PB, MiTg, PL and the Pr5 (Figures 5I–P), whereas *Foxp2* and *Htr1a* expression is more specific to the ILL (Figures 5A–H).

#### 3.2.4. Genes expressed in the ILL and VLL

There were 12 genes expressed in both the ILL and VLL (Table 1): *Hcn1*, Hyperpolarization activated cyclic nucleotide gated potassium channel 1 (Figures 6A–C); *Igfbp5*, Insulin-like growth factor binding protein 5 (Figures 6D–F); *Tnnt1*, Troponin T1 (Figures 6G–I); *Cacng5*, Calcium channel voltage-dependent gamma subunit 5; *Cd24a*, CD24a antigen; *Crtac1*, Cartilage acidic protein 1; *Kcnh7*, Potassium voltage-gated channel subfamily H member 7; *Kcnq4*, Potassium voltage-gated channel subfamily Q member 4; *Meis2*, Meis homeobox 2; *Mgat5b*, Mannoside acetylglucosaminyltransferase 5 isoenzyme B; *Prkcd*, Protein kinase C delta; and *Rims3*, Regulating synaptic membrane exocytosis 3 (data not shown).

*Tnnt1, Cd24a, Crtac1*, and *Kcnq4* (Figures 6G–I and data not shown) are expressed in scattered cells of the ILL and more strongly in the VLL, while *Hcn1*, *Igfbp5, Cacng5, Kcnh7, Meis2, Mgat5b, Prkcd* and *Rims3* are expressed more homogeneously throughout the ILL and VLL (Figures 6A–F and data not shown).

#### 3.2.5. Genes expressed in the VLL

Regarding the most caudal nucleus, 7 genes were only expressed in the VLL (Table 1): *Calb1*, Calbindin 1 (Figures 7A–D); *Doc2g*, Double C2 gamma (Figures 7E–H); *Tmem215*, Transmembrane protein 215 (Figures 7I–L); *Gata3*, GATA binding protein 3; *Kit*, KIT proto-oncogene receptor tyrosine kinase; *Mgll*, Monoglyceride lipase; and *Sfrp1*, Secreted frizzled-related protein 1. In addition, *Calb1* is also expressed in a few sparce neurons in the ILL (data not shown).

### 3.3. Genes in the LLN associated with deafness and hearing disorders

Five of the genes identified were reported to carry mutations that cause hearing-related mouse phenotypes in the Mouse Genome Informatics (MGI) database: *Gata3, Kcnq4*, *Gabra5, Hcn1*, and *Kit*. In the Online Mendelian Inheritance in Man (OMIM) database, 3 human orthologous were associated with deafness or hearing loss (*GATA3, KCNQ4* and *KIT*). From the results of these searches, we discarded other genes whose known hearing phenotypes were related either to ear morphology (*Meis2, Prkcd*) or language learning (*Foxp2*).

There were also 2 genes (*KCNH7* and *TMEM215*) that belong to the DFNA16 (Deafness, autosomal dominant 16: Fukushima et al., 1999) and DFNA47 (Deafness, Autosomal Dominant 47: D’Adamo et al., 2003) loci, respectively, which are critical chromosomal regions associated with deafness whose causative genes are unknown to date.

**FIGURE 8:**
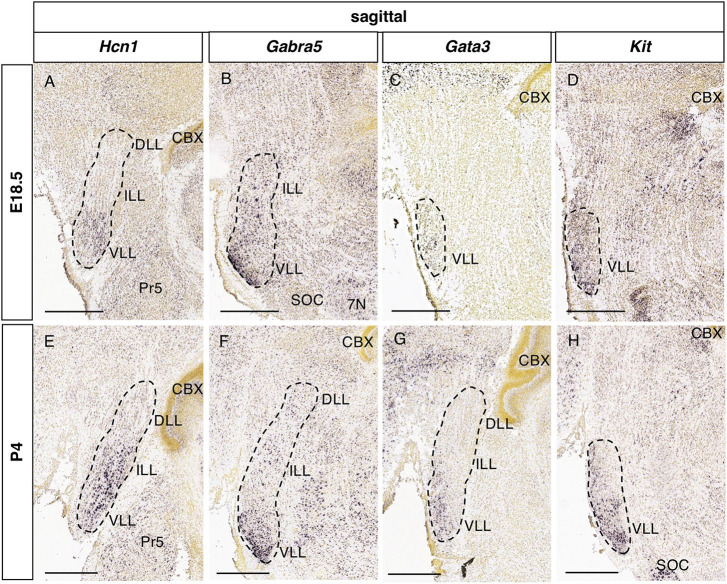
Expression of genes associated with hearing dysfunction at perinatal stages. Brightfield images of sections from E18.5 **(A–D)** and P4 **(E–H)** brains processed to detect *Hcn1* **(A,E)**, *Gabra5* **(B,F)**, *Gata3* **(C,G)**, and *Kit* **(D,H)** expression. Scale bars = 500 μm.

Considering the possible involvement of these genes in congenital hearing dysfunction, we assessed their expression in the lateral lemniscal nuclei at embryonic (E18.5) and early postnatal (P4) stages. In the Allen Developing Mouse Brain Atlas there are available ISH experiments for 4 of these genes (*Hcn1, Gabra5. Gata3* and *Kit*). *Hcn1* expression is restricted to the VLL at E18.5 (Figure 8A), while it is expressed in the ILL and VLL at P4 (Figure 8E), coinciding this early postnatal stage with the adult pattern. In the case of *Gabra5*, it is expressed in the VLL at E18.5 (Figure 8B) and P4 (Figure 8F), so that the additional expression in the DLL that we have described in the adult would appear at later stages. *Gata3* (Figures 8C, G) and *Kit* (Figures 8D, H) are expressed at E18.5 and P4 in the VLL, similarly to adult stages.

## 4. Discussion

We set out here to look for genes differentially expressed across the LLN, identifying 36 genes with discrete expression patterns within this structure. Of these genes, 11 had previously been reported to be expressed in the LLN and thus, we revised their expression pattern relative to previous studies. On the other hand, we provide new data on the regionalized expression of 25 genes. Of these, 4 genes had previously been described in the LLN but without specifying the particular nuclei to which they may be restricted (Table 1).

The results of our screening are consistent with the patterns of expression proposed for the *Pax7* and *Pnoc* genes, both of which are expressed in the DLL (Stoykova and Gruss, 1994; Boom et al., 1999). However, based on the anatomical criteria established here, we propose that *Foxp2*, which has been reported in the DLL (Campbell et al., 2009), is not expressed in this dorsal structure but rather in the ILL. In fact, DLL and ILL misidentification has occurred several times in the literature and indeed, *Math1* (MacHold and Fishell, 2005), *Wnt3a* (Louvi et al., 2007), and *Slc17a6* (VGluT2) (Rose et al., 2009; Di Bonito et al., 2013) labeling in the ILL was misinterpreted as the DLL as noted by Di Bonito and Studer (2017). Similarly, the *Pax7* labeling indicated in the ILL (Moreno-Bravo et al., 2014) corresponds rather to the DLL, at least in the adult brain (present work).

The *Hcn* channel family (*Hcn1-Hcn4*) participates in the excitability of cells in the auditory system (He et al., 2014). Furthermore, these genes are differentially expressed in mice brainstem auditory nuclei (Leao et al., 2006), including the LLN (Caspari et al., 2015). The expression of *Hcn1* has previously been described in the VLL of rats (Koch et al., 2004). However, what was considered to be the VLL in that study included the ILL, probably because the classification followed only considered the DLL and VLL segregation (Malmierca, 2015). Here, we found *Hcn1* to be expressed in the ILL as well as the VLL. Elsewhere, two dorsal and ventral subregions of the VLL were described in mice, based on stronger and weaker *Hcn1* expression, respectively, suggesting different roles in sound processing (Caspari et al., 2015). However, the ISH experiments analyzed in the present work seem to lack sensitivity to discern this gradient, which was witnessed through fluorescent immunohistochemistry. Alternatively, *Hcn1* and the *Kcna1* channel are expressed across the entire LLN in bats, a pattern that may be functionally related to the echolocation characteristic of these species (Pätz et al., 2022).

Calbindin immunoreactivity in neurons appears in the DLL and in a few VLL neurons of adult rats (Friauf, 1994), a pattern distinct to that observed in the mouse where *Calb1* is expressed in the VLL and a few ILL neurons (present work). This latter pattern is on the whole similar to that described in two distant species, the mustached bat (Zettel et al., 1991) and the chinchilla (Kelley et al., 1992), although interspecies differences between bats have been reported (Pätz et al., 2022). These results suggest significant evolutionary differences in the expression of this molecule as previously proposed (Joven et al., 2013), in addition to the variations in Calbindin expression in the brain at different developmental stages including the auditory nuclei (Förster and Illing, 2000).

In general, the VLL and the DLL contain mainly inhibitory neurons (VIAAT/VGAT/*Slc32a1*, Gad67/*Gad1*), whereas the profile of the ILL is mainly excitatory (VGluT1/*Slc17a7*, VGluT2/*Slc17a6*): (Tanaka and Ezure, 2004; Ito et al., 2011, 2015, 2018; Di Bonito and Studer, 2017). Our results are consistent with this neurotransmitter distribution, since we found that *Slc32a1*, *Gad1*, and *Gad2* (GABAergic markers) are expressed in the DLL and VLL, while *Slc17a6* (glutamatergic marker) is expressed in the ILL. In addition, we found that the GABA_*A*_ receptor subunit *Gabra5* is also expressed in the DLL and VLL, along with other subunits reported previously (Campos et al., 2001).

The VLL displays a regional pattern concerning the expression of two genes, *Inhbb* and *Rreb1*, expressed only in the medial but not in the lateral planes. This may be due to the VLL nuclei extending over several rhombomeres (r2-r4), such that the r2 and r3 parts of the VLL are evident in lateral planes whereas the r4 component of this nucleus is mainly seen in medial planes. The regions of expression in the VLL in which these genes might or might not be expressed could correspond to further subdivisions of the rodent VLL (Caspari et al., 2015), as occurs in bats (Ito et al., 2018; Pätz et al., 2022). We named these two subdivisions the rostral and caudal VLL, respectively, following the topological rostrocaudal axis of the brainstem that takes into account the pontine flexure (Puelles et al., 2013).

The division of the LLN into DLL and VLL is based on the existence of two distinct functional systems, a binaural dorsal system and a monoaural ventral system (Malmierca and Ryugo, 2012; Malmierca, 2015). However, the aforementioned VLL can be considered a larger ventral complex that includes the ILL as a separate cell population, distinguishable by criteria of morphology, connectivity and gene expression and neurotransmitter phenotype (Felmy, 2019). In fact, the ILL contains mostly binaural cells so that it is a different entity from the monoaural VLL (Nayagam et al., 2006). Regarding connectivity, the superior olivary complex projects to the ILL but not to the VLL (Kelly et al., 2009). ILL and VLL can be also differentiated according to gene expression (Ito et al., 2011; Di Bonito and Studer, 2017). Additionally, VLL cells constitute an *En1*-lineage derived from r4 (Di Bonito et al., 2013, 2017; Altieri et al., 2016) while ILL is a different cell population derived from an Atoh1/Wnt1/Wnt3a lineage whose precise rhombomeric origin is unknown (MacHold and Fishell, 2005; Wang et al., 2005; Louvi et al., 2007; Rose et al., 2009; Di Bonito and Studer, 2017). In the present work we found 4 genes expressed exclusively in the ILL and 7 genes expressed only in the VLL, so that these 11 genes can be also used to discriminate one nucleus from another. Together with the distinguishable connectivity and morphology of each LL nucleus, these data support the idea that the ILL can be considered a different component from the VLL.

Nevertheless, the homology of this classification of either two or three nuclei in mammals compared to other species (i.e., sauropsids) is not straightforward. In reptiles, a single lemniscal nucleus has been described (Belekhova et al., 2008; Yan et al., 2010; Tang et al., 2012) in contraposition to the three lemniscal nuclei (dorsal, intermediate and ventral) described in birds (Arends and Zeigler, 1986; Wild et al., 2001, 2009). Regarding the GABAergic phenotype in avians, it has been observed in dorsal and ventral parts of the lemniscal complex (Müller, 1987; Carr et al., 1989), while the glutamatergic marker VGLUT2 is similarly found in both dorsal and ventral nuclei (Islam and Atoji, 2008; Karim et al., 2014; Hussan et al., 2022). Therefore, to date a segregation between GABAergic and glutamatergic populations within the avian LLN nuclear complex has not been described, in contrast with mammals. This difference may respond to evolutive variations so that it would be interesting to compare the expression of the other regionalized LLN genes that we have described in this work.

As commented in Results, 3 of these genes (*Gata3, Kcnq4* and *Kit*) are associated to hearing disorders in both mouse and human. *GATA3* is defective in the Barakat syndrome, a disorder that includes sensorineural deafness (Barakat et al., 2018; Michalski and Petit, 2019). *Gata3* is expressed in the lemniscal nuclei at developmental stages (Figure 8), suggesting the possible involvement of these nuclei in this congenital syndrome. *KCNQ4* codes for a potassium channel that is behind DFNA2 non-syndromic hearing loss (Kubisch et al., 1999), although its developmental expression would need to be analyzed. On the other hand, *Kit* displays expression in the LLN at perinatal stages (Figure 8), which may be related to the deafness phenotype associated to its mutation (Ruan et al., 2005). Additionally, this phenotype has been reported in a single clinical case with *KIT* mutation (Spritz and Beighton, 1998).

In addition, two LLN genes are expressed in critical loci associated with deafness, *Kcnh7* in DFNA16 and *Tmem215* in DFNA47. *Kcnh7* is a potassium voltage-gated channel that would be a good candidate to be responsible for DFNA16, like the aforementioned *KCNQ4*, and *KCNQ1* and *KCNE1* potassium channels that when mutated result in Jervell and Lange-Nielsen cardioauditory syndrome (Neyroud et al., 1997; Schulze-Bahr et al., 1997). Concerning, *Tmem215*, it codes for a transmembrane protein involved in the survival of endothelial cells (Liu et al., 2019), although its possible involvement in the auditory system would need to be analyzed.

In summary, here we provide new insights into the regionalization of the LLN, further characterizing the molecular identity of each LL nucleus. This molecular regionalization probably has implications in auditory functions, as suggested by the known hearing phenotypes associated with some of these genes.

## Data availability statement

Publicly available datasets were analyzed in this study. This data can be found in: The Gene Expression Nervous System Atlas (GENSAT) Project, NINDS Contracts N01NS02331 and HHSN271200723701C to the Rockefeller University (New York, NY), available from www.gensat.org; ©2004 Allen Institute for Brain Science, Allen Mouse Brain Atlas, available from mouse.brain-map.org, ©2008 Allen Institute for Brain Science, Allen Developing Mouse Brain Atlas, available from developingmouse.brain-map.org, and ©2014 Allen Institute for Brain Science, Allen Mouse Brain Connectivity Atlas, available from connectivity.brain-map.org.

## Author contributions

PA conceived the research. IG-G, FM, and PA performed the data mining, analyzed the data, prepared the figures, and wrote the manuscript. All authors contributed to the article and approved the final submitted version.

## Footnotes

1
www.gensat.org/

2
connectivity.brain-map.org/

3
mouse.brain-map.org

4
developingmouse.brain-map.org/

5
www.omim.org

6
www.ensembl.org/

7
www.informatics.jax.org/
